# Social determinants of mortality from COVID-19: A simulation study using NHANES

**DOI:** 10.1371/journal.pmed.1003490

**Published:** 2021-01-11

**Authors:** Benjamin Seligman, Maddalena Ferranna, David E. Bloom

**Affiliations:** 1 New England Geriatric Research, Education, and Clinical Center, VA Boston Healthcare System, Boston, Massachusetts, United States of America; 2 Harvard Medical School, Boston, Massachusetts, United States of America; 3 Harvard Center for Population and Development Studies, Cambridge, Massachusetts, United States of America; 4 Department of Global Health and Population, Harvard T.H. Chan School of Public Health, Boston, Massachusetts, United States of America; Universitair Medisch Centrum Utrecht, NETHERLANDS

## Abstract

**Background:**

The COVID-19 epidemic in the United States is widespread, with more than 200,000 deaths reported as of September 23, 2020. While ecological studies show higher burdens of COVID-19 mortality in areas with higher rates of poverty, little is known about social determinants of COVID-19 mortality at the individual level.

**Methods and findings:**

We estimated the proportions of COVID-19 deaths by age, sex, race/ethnicity, and comorbid conditions using their reported univariate proportions among COVID-19 deaths and correlations among these variables in the general population from the 2017–2018 National Health and Nutrition Examination Survey (NHANES). We used these proportions to randomly sample individuals from NHANES. We analyzed the distributions of COVID-19 deaths by race/ethnicity, income, education level, and veteran status. We analyzed the association of these characteristics with mortality by logistic regression. Summary demographics of deaths include mean age 71.6 years, 45.9% female, and 45.1% non-Hispanic white. We found that disproportionate deaths occurred among individuals with nonwhite race/ethnicity (54.8% of deaths, 95% CI 49.0%–59.6%, *p* < 0.001), individuals with income below the median (67.5%, 95% CI 63.4%–71.5%, *p* < 0.001), individuals with less than a high school level of education (25.6%, 95% CI 23.4% –27.9%, *p* < 0.001), and veterans (19.5%, 95% CI 15.8%–23.4%, *p* < 0.001). Except for veteran status, these characteristics are significantly associated with COVID-19 mortality in multiple logistic regression. Limitations include the lack of institutionalized people in the sample (e.g., nursing home residents and incarcerated persons), the need to use comorbidity data collected from outside the US, and the assumption of the same correlations among variables for the noninstitutionalized population and COVID-19 decedents.

**Conclusions:**

Substantial inequalities in COVID-19 mortality are likely, with disproportionate burdens falling on those who are of racial/ethnic minorities, are poor, have less education, and are veterans. Healthcare systems must ensure adequate access to these groups. Public health measures should specifically reach these groups, and data on social determinants should be systematically collected from people with COVID-19.

## Abstract 2

In this simulation study, Benjamin Seligman and colleagues explore socio-demographic factors associated with COVID-19 deaths in the US.

## Author summary

**Why was this study done?:**

- The COVID-19 epidemic in the United States of America (US) has been found to disproportionally affect racial/ethnic minorities and areas with higher concentrations of poverty.
- Few data on COVID-19 deaths with respect to individual-level social determinants of health other than race/ethnicity are available.

**What did the researchers do and find?:**

- We used the 2017–2018 National Health and Nutrition Examination Survey (NHANES) and publicly reported data on COVID-19 deaths from the public health agencies of the US, China, the United Kingdom, Spain, Italy, and France to simulate COVID-19 deaths among noninstitutionalized (e.g., not residing in a prison or nursing home) adults aged 20 years or older in the US.
- We found large social gradients in COVID-19 mortality. Adults from households earning less than the median income made up two-thirds of COVID-19 deaths, while those with less than a high school education accounted for approximately 1 in 4 deaths. Veterans also accounted for nearly 1 in 5 deaths, despite representing less than one-tenth of the population.
- Our simulation reproduced known racial/ethnic disparities in COVID-19 mortality.

**What do these findings mean?:**

- The associations between social determinants of health and COVID-19 mortality are similar in scale to those between hypertension and diabetes and COVID-19 mortality.
- COVID-19 mitigation will require measures to support people from low-income, low-education communities and the healthcare systems that serve them.

## Introduction

The SARS-CoV-2 pandemic is a profound challenge to healthcare systems and societies. While no segment of society is unaffected, some groups face disproportionate burdens of illness. Multiple studies have established the increased risks of severe illness and mortality with age and comorbidity [1–6]. Within the United States of America (US), geographic differences in the prevalence and incidence of COVID-19 morbidity and mortality are known [4]. However, data on social determinants of health and the associated risks of infection with SARS-CoV-2 and death from COVID-19 are less available. US Centers for Disease Control and Prevention (CDC) surveillance has demonstrated the overrepresentation of African Americans among people hospitalized with COVID-19, but more detailed information is lacking in disease statistics [7,8]. Other major sources of COVID-19 statistics likewise lack information on social determinants of health [9,10].

Social determinants of health play an important role in the spread of and mortality from epidemics, from influenza in 1918 to Ebola in 2014 and others, including the first severe acute respiratory syndrome (SARS) epidemic [11–16]. They affect susceptibility to acquiring infection, due to differences in social contacts and differences in living circumstances [15,17–19]. They affect severity of illness, in part through concentration of comorbidity in susceptible groups [20–22]. Finally, they also affect outcomes through differential access to healthcare, which may in turn limit opportunities to identify and contain local outbreaks [17,23–25].

Understanding the COVID-19 burden in terms of social determinants of health is important for policymaking and targeting both public health and clinical interventions. Ecological-level data show that areas with higher poverty rates and larger proportions of individuals who identify as racial/ethnic minorities have higher COVID-19 mortality [26]. However, few analyses to date consider social determinants at the individual level. Here, we use simulation to investigate the distribution of COVID-19 mortality with respect to social determinants of health at an individual level.

## Methods

Because data on social determinants of health among COVID-19 deaths are limited, we simulated mortality. We started with univariate distributions of COVID-19 deaths by age, sex, race/ethnicity, and comorbid conditions—as reported by multiple public health agencies from the spring to summer of 2020—and the correlations among these variables from the 2017–2018 National Health and Nutrition Examination Survey (NHANES). Using these data, we estimated the joint distribution of deaths by these variables. The probabilities obtained from this joint distribution were used as weights for simulating COVID-19 deaths in the NHANES cohort. This provided a sample with which we could investigate social determinants of health in COVID-19 mortality.

### NHANES sample

Data on individuals aged 20 years or older in the general population were taken from the 2017–2018 cycle of NHANES (*n* = 5,265), a nationally representative study of the health of noninstitutionalized Americans [27]. This study comprised a questionnaire, a physical exam, and selected laboratory studies. Questions asked addressed health status, including comorbidities; demographic information; and social determinants of health, such as income and education.

To code the presence of certain comorbidities we used definitions incorporating both questionnaire and examination or laboratory findings. Hypertension was defined as the presence of either self-reported hypertension in the health questionnaire or an average blood pressure greater than 140/90 mm Hg on examination; we did not incorporate use of antihypertensives due to missingness [28,29]. Diabetes was defined as self-report or a hemoglobin A1C greater than 6.5% [30]. Chronic kidney disease (CKD) was defined as self-report of either kidney failure or dialysis or an estimated glomerular filtration rate less than 60 ml/min/1.73 m^2^ using CKD-EPI [31,32]. Ischemic heart disease (IHD) was defined as self-report of coronary heart disease, angina, or heart attack. Chronic obstructive pulmonary disease (COPD) was defined as self-report of emphysema, chronic bronchitis, or COPD. Additionally, we created a comorbidity index based on the number of comorbidities each participant had, including the presence of hypertension, diabetes, CKD, IHD, COPD, and self-reported asthma, congestive heart failure, stroke, liver disease, and cancer.

For social determinants of health, we considered race/ethnicity, income, education, and veteran status based on their availability in NHANES. We specifically considered veterans because they are disproportionately older compared with the general population and frequently face service-related health conditions [33,34]. Cases with missing data or with responses of “don’t know” or “refused” were dropped from the final analysis. For race/ethnicity, the categories “Mexican American” and “Other Hispanic” were combined into “Hispanic.” For income, we used household income, limited analysis to responses within specific income ranges, and did not include the answers “under $20,000” and “$20,000 and over.” In the NHANES data, age was top-coded at 80 years, and income was top-coded at $100,000 per year.

### Simulation of COVID-19 deaths

Simulating COVID-19 deaths involved 3 steps, with the last 2 repeated in each simulation run. This was necessary given the absence of cross-tabulations of COVID-19 deaths by age, sex, and comorbidity, with only univariate distributions consistently available. In the first step, marginal distributions of age, sex, and comorbidity were taken from multiple public health agencies and used to estimate prior distributions. In the second step, the marginal distributions of age, sex, and comorbidity were randomly drawn from the priors. Using the correlations among these variables from NHANES, we then approximated their joint distribution. In the third step, the joint distribution was used to reweight the NHANES sample to represent COVID-19 deaths. We give more detail on each step in the following.

As joint distributions of characteristics of COVID-19 deaths were unavailable, we first obtained their marginal distributions. We considered the marginal distributions of deaths by age, sex, the absence of comorbidity, and presence of each of hypertension, diabetes, CKD, IHD, COPD, and cancer. These were obtained from the CDC, the United Kingdom’s Office for National Statistics, Santé Publique France, Istituto Superiore di Sanità in Italy, Instituto de Salud Carlos III in Spain, and the China Center for Disease Control and Prevention [35–41]; S1 Text provides further details. Comorbidity data from the CDC, the Office for National Statistics, and Santé Publique France were excluded from fitting the model due to their reliance on death certificate data, which underestimate the prevalence of comorbidities compared with reported data from US hospitals and from systematically collected data from Italy, Spain, and China [42,43]. We then fit maximum-likelihood beta or Dirichlet (for age) priors to each marginal distribution. To estimate a joint distribution, race was set as an indicator variable for non-Hispanic white and fixed to the proportion reported by the CDC, as this was the only agency that reported race/ethnicity.

The second step was to approximate the joint distributions of the aforementioned characteristics. To do this, we used marginal distributions for age, sex, and each of the comorbidities drawn from their respective priors, the fixed marginal distribution of non-Hispanic white individuals, and the correlations of these variables from NHANES to estimate a joint distribution using a Gaussian copula [44]. We then assigned the joint probabilities as weights to each NHANES participant such that the participants would, in total, represent 200,000 deaths. We repeated step 2 1,000 times. We checked calibration against CDC-published distributions of deaths by age, by sex, and by race/ethnicity. We report our results and data in terms of gender, which is reported in NHANES. However, data from public health agencies are given in terms of sex.

### Analytic approach

With the simulated data we produced distributions across variables of interest. Differences in proportions were assessed by a bootstrap chi-squared test compared to a null distribution of 1,000 replicates randomly sampled from NHANES. To assess the independent contributions of these variables, we also analyzed the data as a case–control study using multivariable logistic regression. In this instance, the NHANES sample was weighted to represent the general population to serve as controls, and to represent COVID-19 deaths to serve as cases. Confidence intervals were bootstrapped from the 1,000 simulation runs, while *p*-values were bootstrapped through 1,000 replicates of a null. We did not have a prespecified analytic plan.

Neither individually identifiable information nor patient health information was used in this study; all data are publicly available and are described in S1 Text. Analysis was conducted by author BS in R version 4.0.2 using the package GenOrd version 1.4.0 [45,46]. Code used for the simulation is available from https://www.hsph.harvard.edu/pgda/data/.

## Results

**Fig 1:**
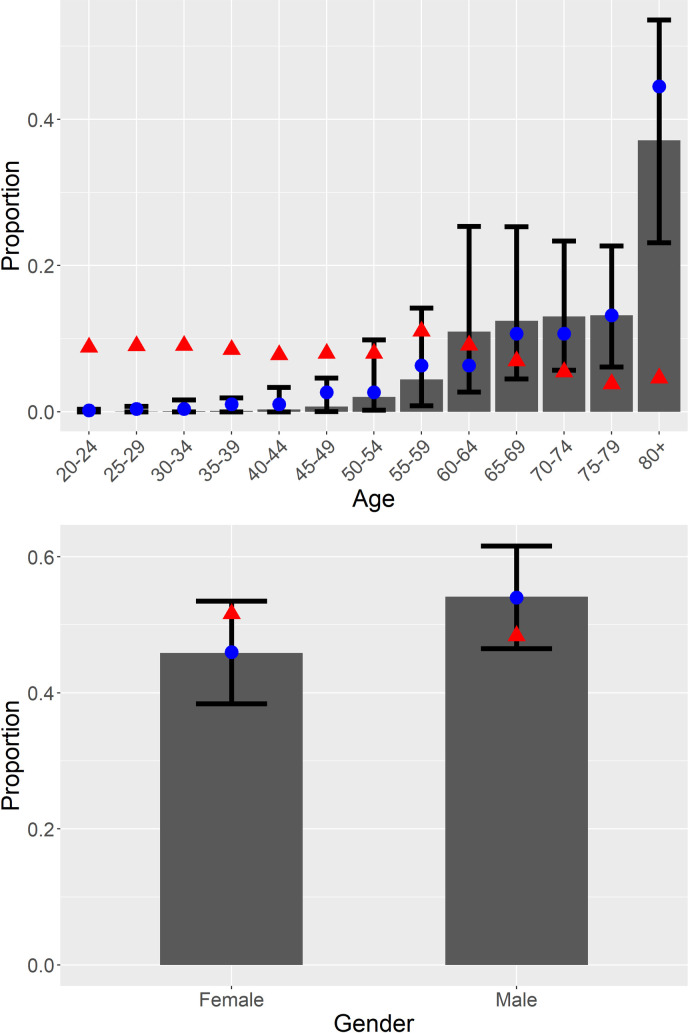
Calibration of simulated distributions of deaths by age and gender against reported distributions from the CDC. Age (top); gender (bottom). Gray bars and whiskers show mean and 2.5th–97.5th percentile range for simulated proportions, the blue circles show the CDC-reported proportions, and the red triangles show the NHANES-estimated proportions for the general population. CDC, US Centers for Disease Control and Prevention; NHANES, National Health and Nutrition Examination Survey.

Table 1 shows the distributions of characteristics of the simulated COVID-19 deaths and the characteristics of their comparison population from NHANES 2017–2018. Fig 1 shows the results of model calibration against age and gender, indicating acceptable calibration of the simulation against the observed age and gender distributions in the US. Clearly, the general pattern of disproportionate deaths among the elderly holds in the simulation, although the simulation indicates a higher proportion of deaths at younger ages than is observed in CDC data. The results for gender show close calibration with observed data and follow the pattern of a greater proportion of deaths among males.

**Table 1:** Characteristics of simulated COVID-19 deaths and the weighted NHANES sample.

Variable	Simulated COVID-19 deaths	CDC-reported COVID-19 deaths	2017–2018 NHANES sample
Age (years)	71.6 (67.7–75.0)	73.2	48.7
Female (%)	45.9 (38.4–53.4)	46.0	51.6
Race/ethnicity (%)			
Non-Hispanic white	45.1 (40.4–51.0)	51.3	63.0
African American	21.8 (18.8–24.4)	21.5	10.7
Hispanic	17.7 (14.8–19.9)	20.6	15.8
Asian American/Pacific Islander	11.5 (10.0–12.9)	4.4	5.6
Other	4.1 (3.2–5.0)	2.2	4.8
Comorbidities (%)			
Hypertension	71.4 (44.4–88.7)		41.4
IHD	4.9 (2.9–7.3)		7.1
Diabetes mellitus	15.5 (9.0–24.4)		14.3
CKD	6.8 (3.1–11.4)		9.1
COPD	2.9 (1.5–4.7)		9.5
Cancer	2.5 (0.2–9.8)		11.2
Income (%)			
$0–$4,999	2.0 (1.6–2.5)		1.8
$5000–$9,999	3.1 (2.6–3.7)		2.0
$10,000–$14,999	8.8 (7.5–10.1)		3.2
$15,000–$19,999	10.4 (9.1–11.8)		4.9
$20,000–$24,999	6.9 (5.7–8.2)		5.0
$25,000–$34,999	17.5 (15.4–20.4)		8.8
$35,000–$44,999	13 (11.2–14.4)		10.8
$45,000–$54,999	5.7 (4.9–6.4)		7.4
$55,000–$64,999	4.2 (3.6–4.9)		6.8
$65,000–$74,999	4.2 (3.5–4.7)		5.6
$75,000–$99,999	7.9 (5.9–10.1)		14.9
≥$100,000	16.3 (14.6–18.2)		29.0
Veteran (%)	19.5 (15.8–23.4)		9.0
Education			
Less than 9th grade	11.0 (9.3–12.6)		3.7
9th–11th grade	14.6 (13.0–16.6)		7.5
High school/GED	23.9 (22.2–25.2)		27.5
Some college or AA	30.4 (28.7–32.1)		30.8
College or above	20.2 (17.9–22.4)		30.6

Values for the simulated COVID-19 sample are presented as the mean (2.5th–97.5th percentiles) from the 1,000 simulation runs. Values for the 2017–2018 NHANES sample are presented using sample weights. Percentages are rounded and may not sum to 100%.

AA, associate’s degree; CDC, US Centers for Disease Control and Prevention; CKD, chronic kidney disease; COPD, chronic obstructive pulmonary disease; GED, General Educational Development; IHD, ischemic heart disease; NHANES, National Health and Nutrition Examination Survey.

**Fig 2:**
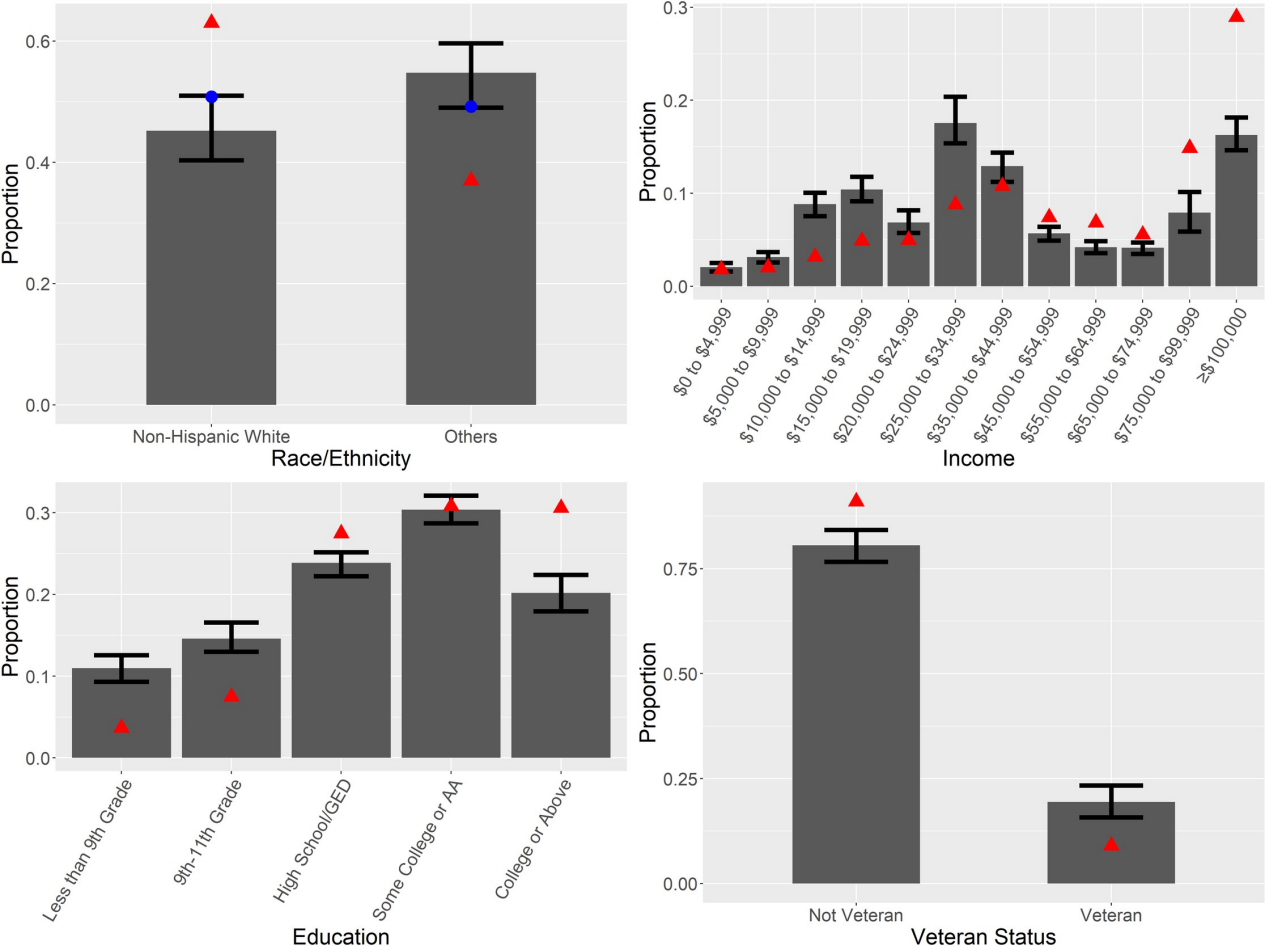
Simulated proportions of deaths by race/ethnicity, income, veteran status, and education level. Panels show mean and 2.5th–97.5th percentile range for the simulations (gray bars and whiskers), the CDC-reported proportions (blue circles, only for race/ethnicity), and the NHANES-estimated proportions for the general population (red triangles). AA, associate’s degree; GED, General Educational Development; NHANES, National Health and Nutrition Examination Survey.

Fig 2 and Table 1 show the distributions of social determinants of health among COVID-19 deaths and in the general population. When we divide the population between non-Hispanic white and all races/ethnicities, the simulation shows that all others are overrepresented among COVID-19 deaths compared with their proportion in the general population (54.8% of deaths, 95% CI 49.0%–59.6%, *p* < 0.001). When data are broken out by specific race/ethnicity (S1 Fig), we see that the simulation captures some disparities, but understates others, particularly among African Americans.

With respect to income, COVID-19 deaths are disproportionately among middle- and lower-income people. Those making below the median income, which is the $55,000–$64,999 category, make up 67.5% of deaths (95% CI 63.4%–71.5%, *p* < 0.001). Similarly, disparities exist regarding level of education: Individuals who have less than a high school level of education are overrepresented among COVID-19 deaths (25.6%, 95% CI 23.4%–27.9%, *p* < 0.001). Disparities with respect to being a veteran are smaller on an absolute scale; however, veterans make up almost 20% of deaths in the simulation (19.5%, 95% CI 15.8%–23.4%, *p* < 0.001) versus 9% of the population.

Table 2 shows the results of the multivariable logistic regression of COVID-19 mortality against age, gender, and the social determinants of health studied. The coefficients show increasing odds of mortality with age, while female gender is associated with lower odds. These data also show increasing odds of mortality for Hispanics, African Americans, and Asian Americans/Pacific Islanders. For income and education, as the level of either one declines, the odds of mortality rise, except for the “some college” category. In this group, relative to high school graduates, the odds of mortality are increased. Being a veteran was not significantly associated with increased odds of death from COVID-19 independent of the other variables considered.

**Table 2:** Odds ratios of death from COVID-19 based on age, gender, race/ethnicity, income, education, and veteran status.

Variable	Odds ratio	95% confidence interval	*p-*Value
Age	1.12	1.09–1.17	<0.001
Female gender	0.64	0.48–0.86	<0.001
Race/ethnicity			
Hispanic	2.56	2.05–3.12	<0.001
African American	3.68	2.84–4.55	<0.001
Asian American/Pacific Islander	4.49	3.55–5.44	<0.001
Other	1.77	1.19–2.39	<0.001
Non-Hispanic white	1	Reference	
Income	0.95	0.94–0.96	<0.001
Education			
<9th grade	1.39	1.26–1.52	<0.001
9th–11th grade	1.32	1.16–1.55	<0.001
High school/GED	1	Reference	
Some college or AA	1.40	1.28–1.55	<0.001
College or above	0.90	0.78–1.08	<0.001
Veteran	1.00	0.98–1.02	0.036

Odds ratios (95% CIs) and *p*-values from multivariable logistic regression of death from COVID-19. Confidence intervals and *p*-values may appear incongruent due to bootstrapping.

AA, associate’s degree; GED, General Educational Development.

## Discussion

Our simulation provides evidence of the scale of social and economic disparities in the COVID-19 epidemic in the US. Mortality from infection disproportionately strikes individuals from low- or middle-income families, individuals with less education, individuals who are of racial/ethnic minorities, and individuals who have served in the military. The disparities identified here are likely underestimates of their true scope. As the simulation only considers age, gender, race/ethnicity, and comorbidity as drivers of death, direct effects of social determinants of health on vulnerability to infection and mortality are not explicitly modeled. These effects include crowded living conditions, limited access to care, and economic hardship that may force people to continue to risk exposure by working. This analysis also does not consider the low wages of many essential jobs that place workers at increased risk of infection, including healthcare workers such as environmental services staff and patient care attendants [47]. Even with these limitations, however, the associations of COVID-19 mortality with social adversity in our study are comparable to the associations of COVID-19 mortality with diabetes (odds ratio 1.75–1.90) and hypertension (relative risk 2.21) [48–51]. Our findings differ from a study that found no difference in income among persons who died or had critical illness during hospitalization for COVID-19 versus those who survived hospitalization without critical illness [52]. This is likely due to our consideration of the general population, versus those who are hospitalized.

Our findings provide further evidence that efforts to reduce COVID-19 mortality should involve prioritizing the needs of disadvantaged communities. This could involve greater assistance for healthcare systems that disproportionately care for low-income or low-education people, such as many public and rural hospital systems, the Veterans Health Administration, and the Indian Health Service. This would also need to involve public health measures, such as paid sick leave, income support, and expansion of health insurance access, to make social distancing more feasible and make care accessible. This may also involve messaging around social distancing and other health behaviors targeted to groups facing social disadvantage. Further, it is crucial that we systematically collect data on social determinants among COVID-19 cases and deaths. These are key data for understanding and controlling the epidemic [53,54].

Our approach has several limitations in addition to not explicitly modeling the effects of social determinants of health. We assume that the correlations among age, gender, race/ethnicity, and comorbidity in the population of individuals who have died from COVID-19 are the same as those in the general population. This is not true in the case of the correlation between gender and age, as males are disproportionately represented in all age groups among COVID-19 deaths, whereas females predominate at older ages in the general population. NHANES data do not include nursing home residents, who make up a large fraction of COVID-19 deaths. This likely contributes to differences between the simulated and observed age distribution of deaths. The other model parameters are based on data from multiple countries, which may affect representativeness for the epidemic in the US, especially with regard to comorbidities, where CDC data could not be used for calibration. Finally, we depend on reliable reporting of deaths, and there is concern that not all COVID-19 deaths are being registered [55,56]. We recommend that further work systematically collect and report data on social determinants of health among individuals affected by COVID-19.

As the Introduction notes, social determinants of health have been associated with disease burden in past epidemics. They have potentially facilitated increased transmission as well, suggesting that control may depend in part on addressing the epidemic specifically among individuals who are poor, have less education, or live in poor conditions [12,14]. As more data become available, we may better understand the roles of particular social determinants and be able to design more effective interventions. By protecting the health of the most vulnerable, such measures could mitigate the toll of the COVID-19 pandemic and protect all Americans.

## Supporting information

S1 Fig Simulated proportions of deaths by race/ethnicity. Figure shows mean and 2.5th–97.5th percentile range for the simulations (gray bars and whiskers), the CDC-reported proportions (blue circles), and the NHANES-estimated proportions for the general population (red triangles). AA, African American; AAPI, Asian American/Pacific Islander; NHW, non-Hispanic white.
(TIF)

S1 Text (DOCX)

## Abbreviations

CDC: US Centers for Disease Control and Prevention
CKD: chronic kidney disease
COPD: chronic obstructive pulmonary disease
IHD: ischemic heart disease
NHANES: National Health and Nutrition Examination Survey
