# Correction: Social determinants of mortality from COVID-19: A simulation study using NHANES

**DOI:** 10.1371/journal.pmed.1003888

**Published:** 2021-12-29

**Authors:** Benjamin Seligman, Maddalena Ferranna, David E. Bloom

In Table 2, the reported results do not perfectly align with results produced by running the posted code. Please see the correct Table 2 here.

**Table 2:** Odds ratios of death from COVID-19 based on age, gender, race/ethnicity, income, education, and veteran status.

Variable	Odds Ratio	95% Confidence Interval	*p*-value
Age	1.12	1.09–1.17	< 0.001
Female Gender	0.63	0.47–0.85	< 0.001
Race/Ethnicity			
Hispanic	2.53	2.03–3.07	< 0.001
AA	3.66	2.82–4.56	< 0.001
AAPI	4.53	3.59–5.50	< 0.001
Other	1.77	1.10–2.38	< 0.001
Non-Hispanic White	1	Reference	
Income (USD, thousands)	0.99	0.99–0.99	< 0.001
Education			
<9th Grade	1.37	1.23–1.51	< 0.001
9th-11th Grade	1.31	1.15–1.55	< 0.001
High School/GED	1	Reference	
Some College	1.37	1.25–1.53	< 0.001
College	0.89	0.77–1.08	< 0.001
Veteran	0.97	0.87–1.12	0.003

Odds ratios (95% CIs) and *p*-values from multivariable logistic regression of death from COVID-19. Confidence intervals and *p*-values may appear incongruent due to bootstrapping.

AA, associate’s degree; GED, General Educational Development

Further, the posted code treats income using ranked categories. Code has been updated to treat income using the mid-points of income categories or the lowest value for the highest category and is available at https://www.hsph.harvard.edu/pgda/data/.
