# Supplementary figures and images for: Social determinants of mortality from COVID-19: A simulation study using NHANES
Source: PLoS Med. 2021 Jan 11;18(1):e1003490. doi: 10.1371/journal.pmed.1003490 (PMC7799807; doi:10.1371/journal.pmed.1003490)

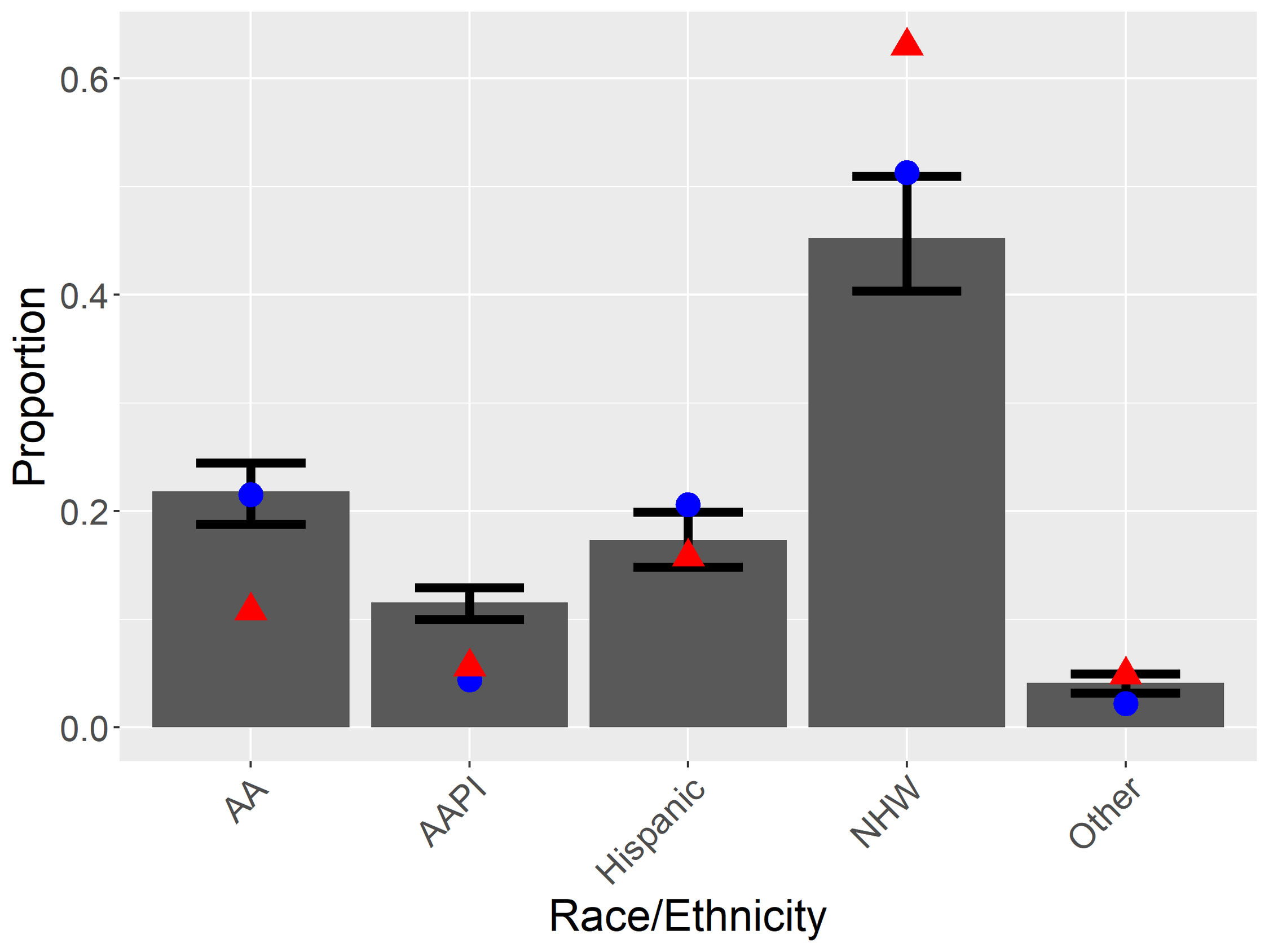

Supplement: S1 Fig — Figure shows mean and 2.5th–97.5th percentile range for the simulations (gray bars and whiskers), the CDC-reported proportions (blue circles), and the NHANES-estimated proportions for the general population (red triangles). AA, African American; AAPI, Asian American/Pacific Islander; NHW, non-Hispanic white. (TIF) [file pmed.1003490.s001.tif]
