# Supplementary material for: Social determinants of mortality from COVID-19: A simulation study using NHANES
Source: PLoS Med. 2021 Jan 11;18(1):e1003490. doi: 10.1371/journal.pmed.1003490 (PMC7799807; doi:10.1371/journal.pmed.1003490)
Supplement: S1 Text — (DOCX) [file pmed.1003490.s002.docx]

# Supplemental Appendix: Data Extraction from Public Health Reports

China:

Data are from <http://journal.yiigle.com/LinkIn.do?linkin_type=pubmed&DOI=10.3760/cma.j.issn.0254-6450.2020.02.003>.

For the age and sex distributions, proportions were taken for individuals aged 20+ in table 1. 10-year age groups were divided evenly into 5-year age groups.

Comorbidity distributions were also taken from table 1.

England & Wales:

Data are from <https://www.ons.gov.uk/releases/deathsinvolvingcovid19englandandwalesjune2020>.

For the age and sex distributions, proportions were taken for individuals aged 20+ in England and Wales in table 2, summed across all months.

For the comorbidity distributions, data were taken from table 5 for England and Wales. Proportions were obtained using case counts divided by the total number of cases given in the table.

France:

Data are from <https://www.santepubliquefrance.fr/maladies-et-traumatismes/maladies-et-infections-respiratoires/infection-a-coronavirus/documents/bulletin-national/covid-19-point-epidemiologique-du-3-septembre-2020>.

For age and sex distributions, proportions were taken for individuals aged 15+ in table 6 for all deaths. For the 15-44 age group, deaths were divided evenly among ages 20-44 assuming no deaths among those under the age of 20.

Comorbidity proportions were taken from the same table.

Italy:

Data are from <https://www.epicentro.iss.it/en/coronavirus/bollettino/Report-COVID-2019_7_september_2020.pdf>.

For age and sex distributions, proportions were taken for individuals aged 20+ in Figure 2. 10-year age groups were divided evenly into 5-year age groups.

Comorbidity proportions were taken from table 2.

Spain:

Data are from <https://www.isciii.es/QueHacemos/Servicios/VigilanciaSaludPublicaRENAVE/EnfermedadesTransmisibles/Documents/INFORMES/Informes%20COVID-19/Informe%20n%C2%BA%2032.%20Situaci%C3%B3n%20de%20COVID-19%20en%20Espa%C3%B1a%20a%2021%20de%20mayo%20de%202020.pdf>.

We used this report as subsequent reports focus solely on cases after 5/10/2020 and do not report detailed information on the sex and comorbidities of deaths.

For age and sex distributions, proportions were taken for individuals aged 15+ in tables 4.1 and 4.3. Those in the 15-29 age group were divided evenly among ages 20-29, assuming no deaths among those under the age of 20.

Comorbidity proportions were taken from table 7.

United States:

Data are from the version of <https://www.cdc.gov/nchs/nvss/vsrr/covid_weekly/index.htm> available as of 9/9/2020 with data as of 9/2/2020.

For age and sex distributions, proportions were taken for individuals aged 15+ in table 1. Those in the 15-24 age group were all assigned to the 20-24 age group in our model, assuming no deaths among those under the age of 20. Four deaths of unknown sex were evenly divided between males and females in their appropriate age categories. There were no deaths reported of unknown age.

Race/Ethnicity proportions were taken from <https://www.cdc.gov/nchs/nvss/vsrr/covid19/health_disparities.htm> table 1.

Comorbidities were taken as proportions based on data from table 3.

| Variable | United States | England +  Wales | France | Spain | Italy | China |
| --- | --- | --- | --- | --- | --- | --- |
| Male | 0.5400572 | 0.5525094 | 0.54 | 0.5652894 | 0.5738793 | 0.638 |
| Age |  |  |  |  |  |  |
| 20-24 | 0.001759469 | 0.0004280364 | 0.001832621 | 0.0006821282 | 0.0002109230 | 0.003503504 |
| 25-29 | 0.003864967 | 0.0010058855 | 0.001832621 | 0.0006821282 | 0.0002109230 | 0.003503504 |
| 30-34 | 0.003864967 | 0.0016265383 | 0.001832621 | 0.0015347885 | 0.0009561843 | 0.009009009 |
| 35-39 | 0.010116946 | 0.0024826110 | 0.001832621 | 0.0015347885 | 0.0009561843 | 0.009009009 |
| 40-44 | 0.010116946 | 0.0046227929 | 0.001832621 | 0.0052864939 | 0.0043871984 | 0.018518519 |
| 45-49 | 0.026529858 | 0.0088175495 | 0.021969631 | 0.0052864939 | 0.0043871984 | 0.018518519 |
| 50-54 | 0.026529858 | 0.0165222044 | 0.021969631 | 0.0159082050 | 0.0174503628 | 0.063563564 |
| 55-59 | 0.063200122 | 0.0284430177 | 0.021969631 | 0.0159082050 | 0.0174503628 | 0.063563564 |
| 60-64 | 0.063200122 | 0.0412841091 | 0.021969631 | 0.0443383356 | 0.0502137353 | 0.151151151 |
| 65-69 | 0.106840815 | 0.0545960407 | 0.078235448 | 0.0443383356 | 0.0502137353 | 0.151151151 |
| 70-74 | 0.106840815 | 0.0911289460 | 0.078235448 | 0.1189826545 | 0.1307441363 | 0.152652653 |
| 75-79 | 0.132024679 | 0.1324558587 | 0.373243739 | 0.1189826545 | 0.1307441363 | 0.152652653 |
| 80+ | 0.445110436 | 0.6165864098 | 0.373243739 | 0.6255603196 | 0.5920749198 | 0.203203203 |
| Comorbidities |  |  |  |  |  |  |
| HTN | 0.2072778 | 0.019 | 0.1622306 | --- | 0.662 | 0.397 |
| IHD | 0.1044631 | --- | --- | --- | 0.277 | 0.227 |
| CKD | --- | --- | 0.08211886 | --- | 0.203 | --- |
| DM | 0.1463549 | 0.025 | 0.1067283 | 0.33 | 0.298 | 0.197 |
| Cancer | 0.04395641 | --- | --- | --- | 0.161 | 0.015 |
| COPD | --- | 0.081 | --- | --- | 0.168 | --- |
| None | --- | 0.089 | 0.34 | 0.05 | 0.04 | 0.328 |

**Table A:** Univariate proportions of age, sex, and comorbidities extracted from reports of COVID-19 mortality. HTN = Hypertension, IHD = Ischemic Heart Disease, CKD = Chronic Kidney Disease, DM = Diabetes Mellitus, COPD = Chronic Obstructive Pulmonary Disease.
